# RNA m1A Methyltransferase TRMT6 Predicts Poorer Prognosis and Promotes Malignant Behavior in Glioma

**DOI:** 10.3389/fmolb.2021.692130

**Published:** 2021-09-22

**Authors:** Beibei Wang, Lihua Niu, Zhengyang Wang, Zhihua Zhao

**Affiliations:** ^1^ Pathology Department, The First Affiliated Hospital of Zhengzhou University, Zhengzhou, China; ^2^ Pathology Department, The Second Affiliated Hospital of Zhengzhou University, Zhengzhou, China

**Keywords:** glioma, m1A regulators, TRMT6, malignant behavior, prognosis

## Abstract

**Background:** Glioma is the most prevalent central nervous system tumor in humans, and its prognosis remains unsatisfactory due to a lack of effective therapeutic targets. The ectopic expression of N1-methyladenosine (m1A) regulators is a key participant in tumorigenesis and progression. However, the m1A regulator expression status, prognostic value, and relationship with tumor clinical features in glioma remain unclear.

**Methods:** Public datasets were used to analyze the mRNA and protein expression levels of m1A regulators. Kaplan–Meier and Cox regression analyses were performed to confirm the prognostic value of m1A regulators in glioma. Cellular experiments were conducted to verify the effect of TRMT6 on cell function. A comprehensive bioinformatics analysis was conducted to identify the potential molecular mechanisms regulated by TEMT6 in glioma.

**Results:** We found that the dysregulation of m1A regulators was closely associated with tumorigenesis and progression in glioma. Furthermore, TRMT6 might be a powerful and independent biomarker for prognosis in glioma. Our study showed that inhibition of TRMT6 suppressed the proliferation, migration, and invasion of glioma cells. Mechanistically, TRMT6 may be involved in glioma progression by regulating cell cycle, PI3K-AKT, TGF-beta, MTORC1, NOTCH, and MYC pathways.

**Conclusions:** Variation in m1A regulators was closely associated with malignant progression in glioma. Silencing TRMT6 suppressed the cell proliferation, migration, and invasion in glioma. m1A regulators, especially TRMT6, might play an essential role in the malignant progression of glioma.

## Introduction

Glioma is the most common central nervous system tumor in humans, accounting for ∼40% of all brain cancers (Dawson and Kouzarides, 2012; Gusyatiner and Hegi, 2018). Glioma is divided into four grades (I-IV), among which grade III and grade IV have close relationships with poor prognosis (Lapointe et al., 2018). Although some progress has been made in the use of surgery and chemotherapy to treat glioma, the 5-year overall survival rate of glioma patients is only 10% (Buckner et al., 2017; Reifenberger et al., 2017). For these reasons, a more in-depth study is urgently needed to understand the potential mechanisms of the tumorigenesis and progression of glioma. Moreover, a new therapeutic target for improving clinical outcomes is critical (Brandsma et al., 2008; Brown et al., 2016).

Recent studies have shown that RNA methylation plays an important role in the determination of cell fate decisions, cell cycle regulation, and cancer development (Gilbert et al., 2016; Zhao et al., 2017; Huang et al., 2020). This indicates that RNA methylation might be a novel and potential therapeutic target for exploration. N1-methyladenosine (m1A) is an important post-transcriptional modification, which could add a methyl group at the N1 position of adenosine and markedly affect RNA structure or protein–RNA interactions (Zhang and Jia, 2018; Shi et al., 2020). Some researchers have designated that m1A is strongly enriched in 5′UTR of tRNA, rRNA, mRNA, and mitochondrial transcripts (Xiong et al., 2018; Zhang and Jia, 2018). Further studies indicate that m1A plays a crucial role in various cellular processes by regulating RNA stability and translation (Zhao et al., 2017; Xiong et al., 2018). m1A methylation involves a series of enzymes which are categorized into three types: m1A methyltransferases also called “writer” (TRMT10C, TRMT61B, TRMT6, and TRMT61A), “‘eraser” are demethylases including ALKBH1 and ALKBH3, and m1A is recognized by m1A-binding protein, and they are jargonized as “reader” (YTHDF1, YTHDF2, YTHDF3, and YTHDC1) (Chujo and Suzuki, 2012; Safra et al., 2017; Dai et al., 2018; Chen et al., 2019). Previous studies have revealed that dysregulation of m1A was closely associated with various human diseases, such as cardiovascular diseases, pulmonary hypertension, and psychiatric disorders (Engel and Chen, 2018; Vissers et al., 2020; Komal et al., 2021; Swietlik et al., 2021). Additionally, some studies have shown that a correlation exists between m1A variation and human tumors. Tasaki et al. found that upregulated ALKBH3 promoted cell proliferation, migration, and invasion in lung cancer (Tasaki et al., 2011). Another study proved that dysregulated m1A regulators were involved in tumor malignant progression via the mTOR and ErbB pathways in gastrointestinal cancer (Zhao et al., 2019). Wang et al. demonstrated that overexpression of TRMT6 was closely associated with poor clinical outcomes in hepatocarcinoma (Wang et al., 2019). As for glioma, Macari et al. found that the activation of TRMT6/61 would promote malignant transformation and progression via sustain tRNA in methylation status in glioma (Macari et al., 2016). However, the clinical value, biological functions, and potential mechanism of m1A regulators in glioma remain unclear.

In this study, we systematically explored the association among the expression of m1A regulators, clinical characters, and prognosis. Then we found that overexpression of TRMT6 was markedly related to poor clinical outcomes in glioma. Furthermore, we demonstrated that the depletion of TRMT6 significantly impaired glioma cell colony formation, proliferation, migration, and invasion. Mechanistically, cell cycle, PI3K-AKT, TGF-beta, MTORC1, NOTCH, and MYC pathways were significantly enriched in high–TRMT6 expression patients. In summary, our results showed that the m1A regulators, TRMT6 in particular, play important roles in the malignant progression of glioma.

## Materials and Methods

### The Datasets Acquisition

The RNA-seq transcriptome data and corresponding clinicopathologic information of glioma were downloaded from The Cancer Genome Atlas (TCGA, https://tcga-data.nci.nih.gov/tcga) and the Chinese Glioma Genome Atlas (CGGA, http://www.cgga.org.cn). We used the Human Protein Atlas (HPA, http://www.proteinatlas.org) to explore the expression level of m1A regulators in normal and glioma tissues. The characteristics of all datasets are presented in Supplementary Table S1.

### Cell Culture

The U251 and U87 cell lines were purchased from the American Type Culture Collection (ATCC, Manassas, VA, United States). They were cultured and maintained in Dulbecco’s modified Eagle medium (DMEM) supplemented with 11% fetal bovine serum (FBS), 100 U/ml penicillin, and 100 µg/ml streptomycin (Gibco, Grand Island, NY, United States). Cells were incubated in a humidified chamber at 37°C in a 95% O2 and 5% CO2 atmosphere.

### Cell Transfection

TRMT6 siRNAs and the negative control (NC) were purchased from GenePharma (Shanghai, China). Cells were transfected using Lipofectamine 3000 (Thermo Fisher, CA, United States) according to the manufacturer’s protocols. The transfection efficiency was verified after 48–72 h. The scrambled sequence was designed as NC siRNA. The sequence of TRMT6 siRNAs is presented in Supplementary Table S2.

### Quantitative Polymerase Chain Reaction

Total RNA was extracted using TRIzol reagent (Life Technologies, CA, United States). cDNA was generated using the SuperScript III First-Strand Synthesis System. qRT-PCRs were performed using the PowerUp SYBR Green kit (ABI, Foster City, CA, United States), and qRT-PCR was performed using the six System (ABI, Foster City, CA, United States). The relative gene expression was calculated using the 2^−ΔΔCt^ method. The sequence of primers is presented in Supplementary Table S2.

### Cell Proliferation Experiments

The proliferation ability of glioma cells was evaluated by a CCK-8 Kit (Dojindo, Japan). We used an EdU staining assay (Olympus Corporation, Tokyo, Japan) to evaluate the DNA synthesis rate of glioma cells. U87 and U251 cell lines were seeded in 6-well plates at a density of 500–1,000 cells per well. After 2 weeks of incubation, colonies were fixed with methanol and stained with 0.4% crystal violet in absolute ethanol, and 50 or more cells were counted.

### Wound Healing and Transwell Assays

Wound healing experiments were conducted to evaluate the migration capability of cells. Cells were inoculated on 6-well plates and allowed to grow from 80 to 90% confluence. Then, cell monolayers were wounded by a 200-µl pipette tip. The migration distance of the cells was quantified at 0 and 48 h. Migration experiments were performed in 24-well Transwell Matrigel invasion chambers (8 μm pores; BD Biosciences) based on the manufacturer’s instructions. In general, cells were starved for 12 h, and then 4.5×10^4^ cells were inoculated into the upper chambers. The lower chambers were filled with DMEM with 15% FBS. After 1 day, the migrated cells were counted.

### Pathway Enrichment Analysis

The biological behaviors of TRMT6 were conducted via Metascape (http://metascape.org/). Gene set variation analysis (GSVA) was performed using the Bioconductor R package “GSVA” to export the potential mechanism of TRMT6 in glioma (Hänzelmann et al., 2013). Furthermore, Kyoto Encyclopedia of Genes and Genomes (KEGG) and Gene Ontology (GO) pathway analyses were conducted using gene set enrichment analysis (GSEA) with clusterProfiler, an R/Bioconductor package (Subramanian et al., 2005).

### Statistical Analysis

All statistical analyses were performed by R (version 3.5.3). Student’s t-test (unpaired and two-tailed) was used to evaluate the differences between two independent groups. In Kaplan–Meier survival analysis, the package of “survminer” was used to determine the best cutoff value of m1A regulators. Patients were divided into two groups (high vs low expression) based on the best cutoff values of m1A regulator expression (smallest *p*-value). Meanwhile, the number of each group was not inferior to 30% of total patients. Cox regression analysis of univariate and multivariate variables was used to determine the relationship between the different variables and survival. In all cases, *p* < 0.05 was considered statistically significant.

## Results

### Correlation Between the Dysregulation of m1A Regulators and Clinical Features in Glioma

**FIGURE 1:**
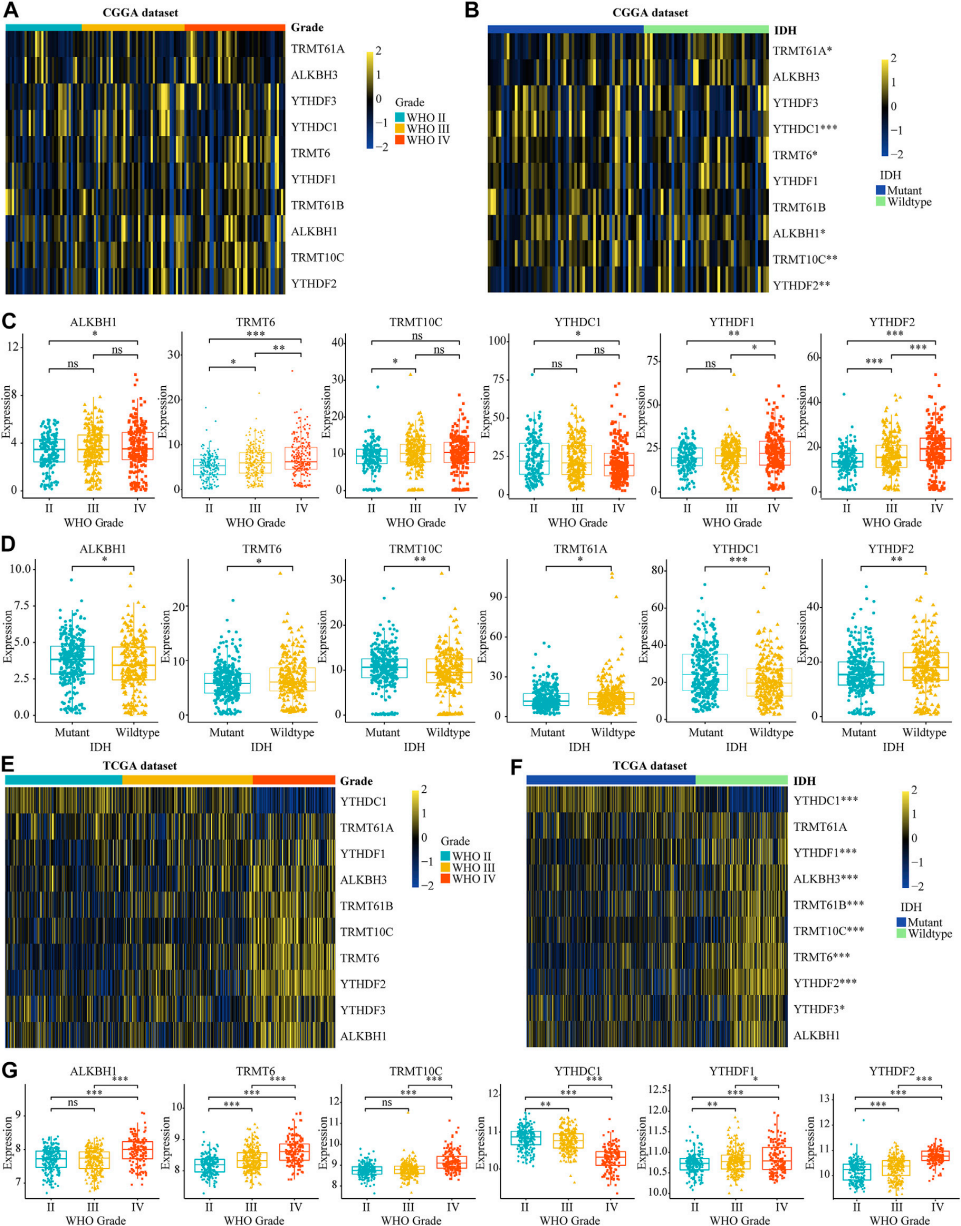
Relationship between the variation of m5C regulators and clinical features in glioma. **(A, B)** The differential expression of m1A regulators with different WHO grades and IDH mutation statuses in CGGA dataset. **(C, D)** The significant differential expression m1A regulators stratified by WHO grade and IDH mutation statuses in CGGA dataset. **(E, G)** The differential expression of m1A regulators stratified by WHO grade and IDH mutation statuses in glioma according to TCGA database. *p < 0.05, **p < 0.01, ***p < 0.001.

To investigate the role of m1A modifications in tumor occurrence and progression for glioma, we systematically analyzed the relationship between the expression of m1A regulators and the clinical features in gliomas, such as WHO grades, 1p19q codeletion, and IDH mutant status according to TCGA and CGGA datasets. The differential analysis showed that ALKBH1, TRMT6, TRMT10C, YTHDF1, and YTHDF2 were significantly overexpressed in high-grade gliomas. Meanwhile, the expression level of YTHDC1 was dramatically decreased in high-grade gliomas (Figures 1A,C,E,G). Additionally, we found that the mRNA expression of TRMT6 and YTHDF2 was higher in IDH wild-type patients and the overexpression of YTHDC1 was significantly related to IDH mutation (Figures 1B,D,F). Further analysis demonstrated that ALKBH1, TRMT6, and YTHDF2 were significantly upregulated in 1p19q non-codel patients (Supplementary Figures S1A–D). Subsequently, IHC images selected from the HPA database also proved that TRMT6, TRMT61B, and YTHDF2 were upregulated in glioma tissues (Supplementary Figure S2). In conclusion, these results suggested that dysregulated m1A regulators were associated with tumorigenesis and progression in glioma.

### The Prognostic Value of the m1A Regulators, Especially TRMT6, in Glioma

**FIGURE 2:**
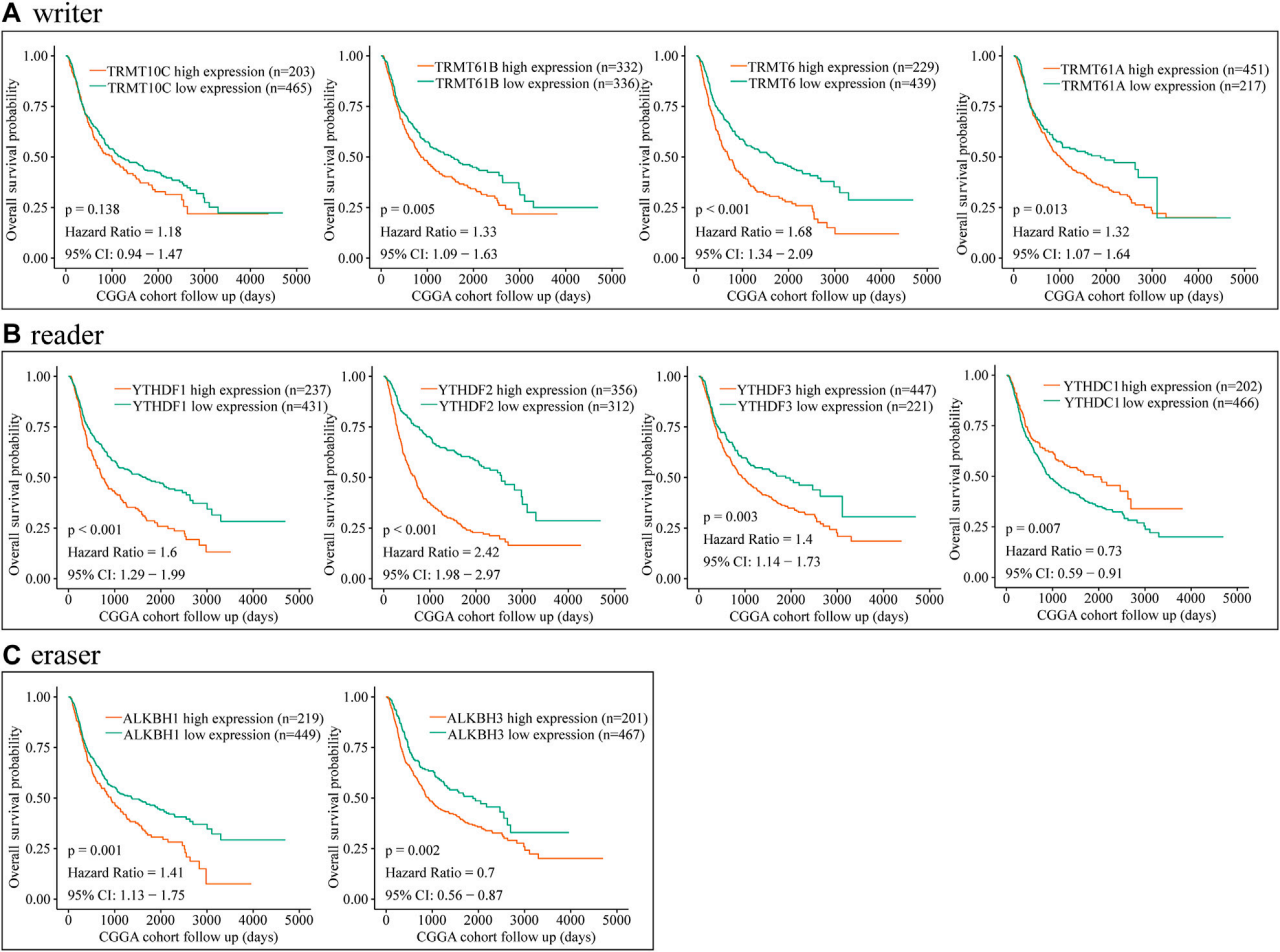
Prognostic value of the m1A regulators in glioma according to CGGA database. **(A–C)** Kaplan–Meier analysis showing the correlation between m1A “writers” expression levels and the glioma patients overall survival rates according to CGGA database.

**FIGURE 3:**
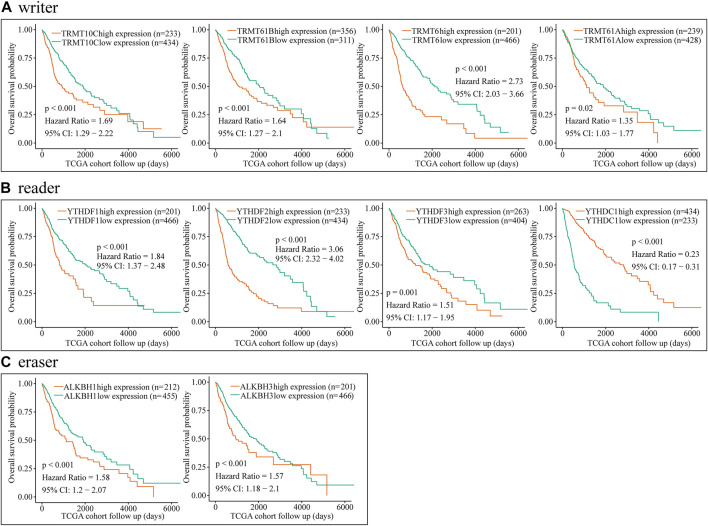
Prognostic value of the m1A regulators in glioma based on TCGA dataset. **(A–C)** Kaplan–Meier analysis showing the correlation between m1A “writers,” “readers,” and “erasers” expression levels and the glioma patients overall survival rates based on TCGA dataset.

To further explore the relationship between dysregulated m1A regulator and glioma prognosis, glioma patients were divided into high- and low-expression groups based on the best cutoff value. Then, we conducted the survival analysis of each m1A regulator independently via CGGA and TCGA databases. Kaplan–Meier survival curves showed that overexpression of TRMT61B, TRMT6, TRMT61A, YTHDFs, ALKBH1, and ALKBH3 was significantly associated with poor overall survival in glioma. Meanwhile, YTHDC1 was a potential protective factor in glioma (Figure 2 and Figure 3). Additionally, correlation analysis showed that there were high correlations among m1A regulators, in particular between YTHDF2 and TRMT6 (Supplementary Figures S3A–D). Previous studies have identified YTHDF2 as a cancer-promoting gene in glioma (Chai et al., 2021). Moreover, subgroup survival analysis indicated that overexpression of TRMT6 was closely associated with poorer prognosis in both YTHDF2 low- and high-expression patients (Supplementary Figures S3E–H).

**FIGURE 4:**
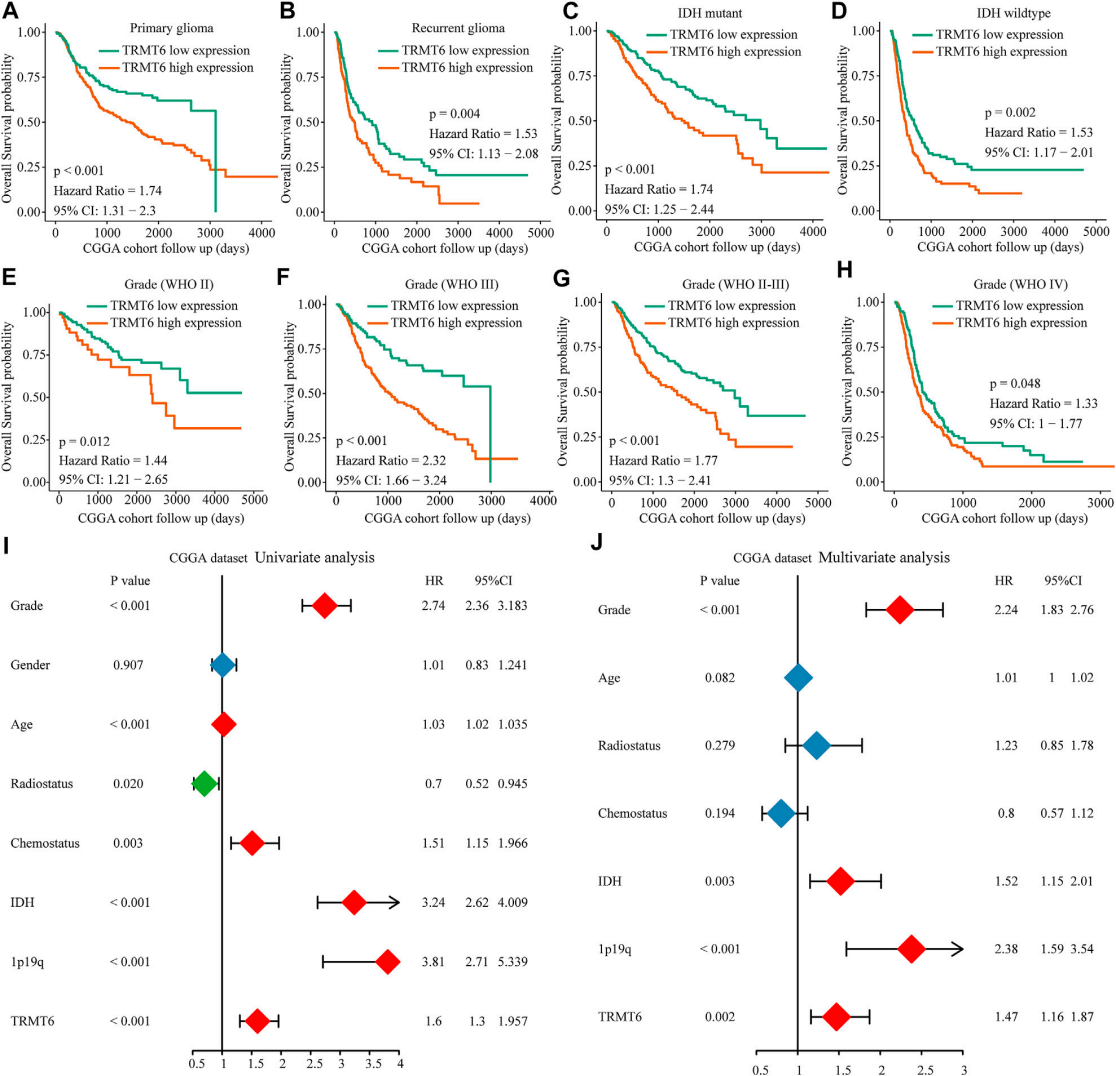
Prognostic value of the TRMT6 in glioma. **(A–H)** Kaplan–Meier overall survival curves of TRMT6 for patients in the CGGA dataset stratified by recurrence, WHO grade and IDH mutation statuses. **(I–J)** Univariate and multivariate Cox regression analyses showed that TRMT6 was an independent prognostic risk factor in CGGA dataset.

In view of TRMT6, the m1A writer was closely associated with clinical progression and poor overall survival in glioma; we further explore the prognosis value of TRMT6. Results showed that overexpression of TRMT6 was closely associated with worse OS in different glioma clinical subtypes such as WHO-grade subtypes, IDH mutation status, and tumor recurrence status (Figures 4A–H). Then, univariate and multivariate Cox regression analyses were performed with CGGA datasets. TRMT6, WHO grade, IDH, and 1p/19q codel status were independent prognostic factors for glioma outcome (Figures 4I,J). In summary, these results confirmed that TRMT6 might be a powerful biomarker for evaluating prognosis in glioma.

### TRMT6 Silencing Inhibits Glioma Cell Proliferation, Migration, and Invasion

**FIGURE 5:**
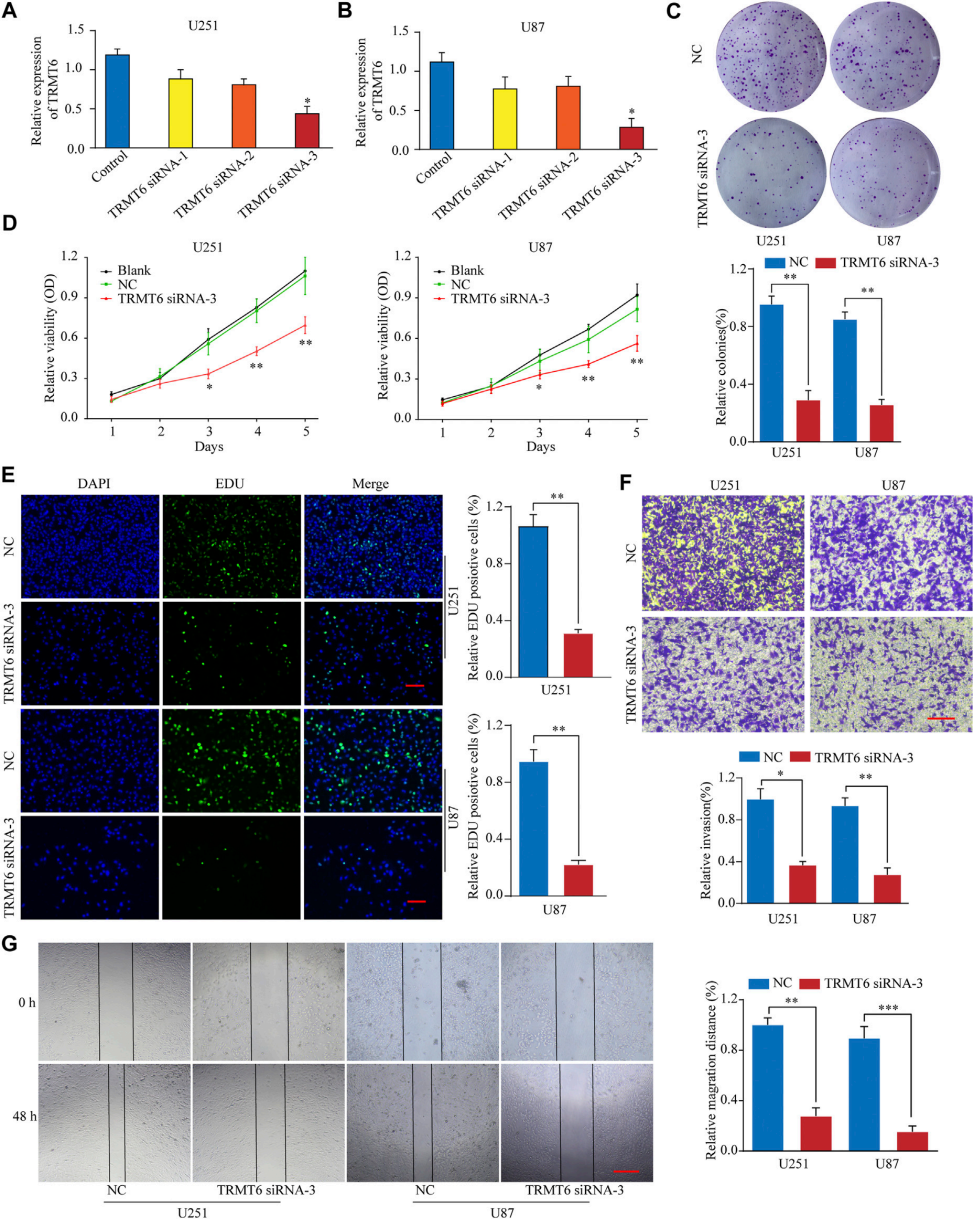
TRMT6 silencing inhibits glioma cell proliferation, migration, and invasion. **(A, B)**: qRT-PCR analysis of TRMT6 expression in glioma cells after transfection with control or TRMT6 siRNAs. **(C)**: Knockdown of TRMT6 dramatically inhibited the cell colony formation ability. **(D)**: Cell proliferation of U251 or U87 cells transfected with NC or TRMT6 siRNA was analyzed by CCK-8 assay. **(E)**: Cell proliferation of U251 or U87 cells transfected with NC or TRMT6 siRNA was analyzed by EdU staining assay. Scale bar, 50 μm. **(F)**: Decreased TRMT6 inhibited U251 or U87 cell invasion ability. Scale bar, 50 μm. **(G)**: Decreased TRMT6 inhibited U251 or U87 cell migration ability. Scale bar, 50 μm *p < 0.05, **p < 0.01, ***p < 0.001.

To explore the functional role of TRMT6 in the malignant behavior of glioma cells, three different siRNAs targeting TRMT6 (siRNA-1, siRNA-2, and siRNA-3) were used. *In vitro* experiments showed that TRMT6 siRNA-3 significantly decreased the expression of TRMT6 in the U251 and U87 cell lines (Figures 5A–B). A colony formation assay demonstrated that glioma cells treated with TRMT6 siRNA-3 generated fewer colonies than the NC group (Figure 5C). A CCK-8 assay showed that TRMT6-siRNA could suppress the growth in glioma cells (Figure 5D). In addition, to examine the proliferation ability of glioma cells, we conducted an EdU assay, which showed that glioma cell proliferation was suppressed after the inhibition of TRMT6 (Figure 5E). To investigate the migration ability of cells after transfection with TRMT6-siRNA-3, we performed a wound healing assay and found marked suppression of cell migration in glioma cells (Figure 5G). The wound healing experiment’s results prompted us to evaluate the invasive potential of glioma cells, so we performed a Transwell assay experiment and found that suppression of TRMT6 could decrease the invasive capability of glioma cells compared to the NC group (Figure 4F). In summary, our data reveal that glioma cell proliferation, migration, and invasion were markedly decreased after TRMT6 silencing.

### Biological Functions Might Be Regulated by TRMT6 in Glioma

**FIGURE 6:**
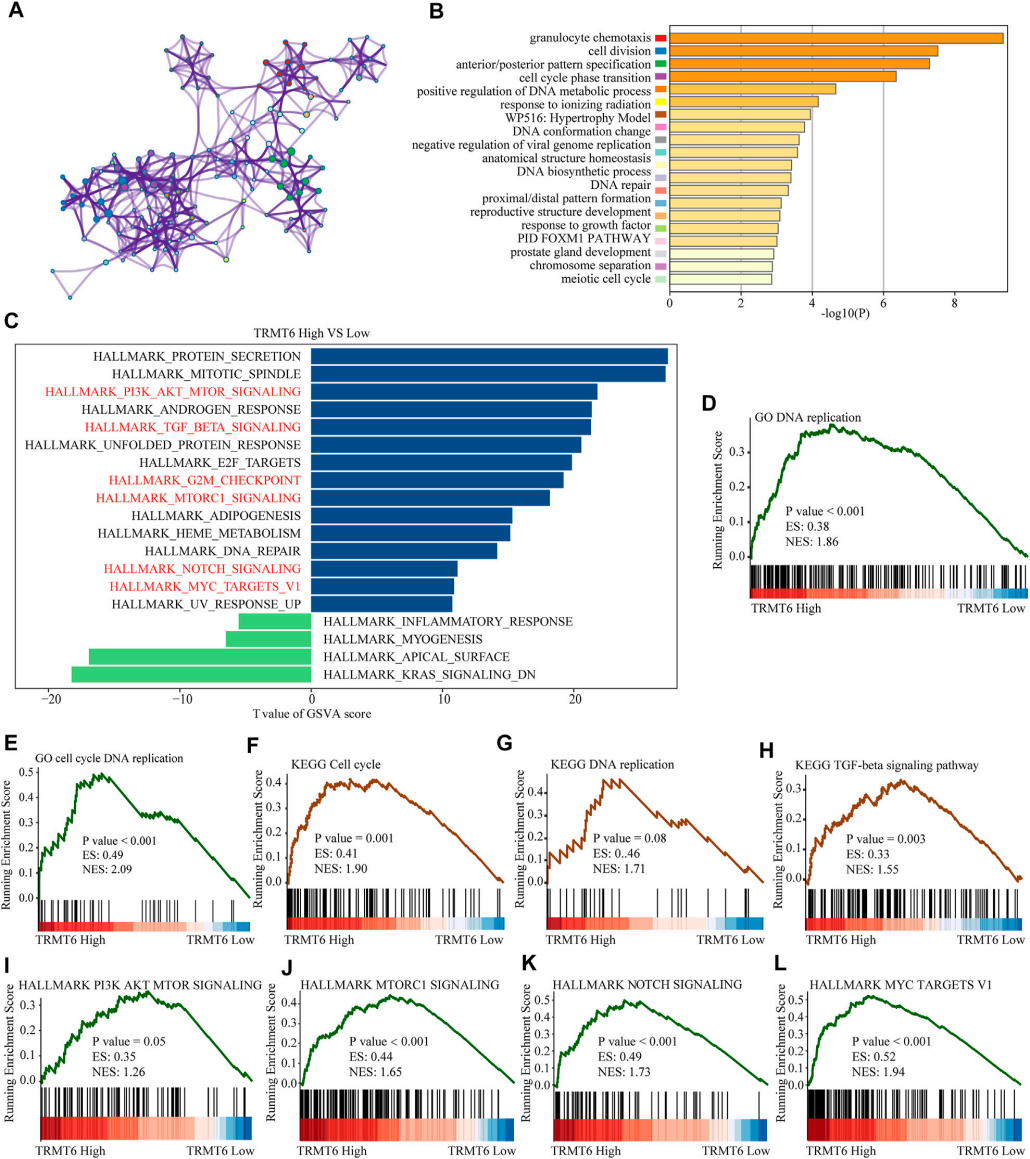
Functional annotation of TRMT6 in glioma. **(A,B)** The network and bar chart of the top 20 significantly enriched biological pathways of upregulated genes in patients with high TRMT6 expression. Each enriched node is presented in a different color. **(C)** GSVA between high– and low–TRMT6 expression patients using hallmark gene sets. **(D–H)** GSEA revealed that cell cycle, PI3K-AKT, TGF-beta, MTORC1, NOTCH, and MYC pathways were significantly enriched in patients with high TRMT6 expression.

To further explore the underlying mechanism of the oncogenic effect of TRMT6 in glioma, we further explored whether patients with different TRMT6 expression statuses had different biological behaviors. The top 200 most significantly upregulated genes of patients with high TRMT6 expression based on the *p*-value were selected to annotate the biological function. The results showed that the inflammatory signaling pathway (granulocyte chemotaxis and PID FOXM1 pathways) and cell cycle pathway (cell division, meiotic cell cycle, chromosome separation, and DNA biosynthetic processes) were significantly enriched in TRMT6-upregulated patients. The top 20 significantly enriched biological pathways are presented in Figures 6A–B. Similarly, GSVA and GSEA analyses further indicated that the cell cycle, PI3K-AKT, TGF-beta, MTORC1, NOTCH, and MYC pathways were significantly enriched in high–TRMT6 expression patients (Figures 6C–L). Overall, these findings showed that TRMT6 might be involved in glioma progression by regulating cell cycle, PI3K-AKT, TGF-beta, MTORC1, NOTCH, and MYC pathways.

## Discussion

Glioma is the most common type of malignant central nervous system tumor. Although the diagnosis and therapy for glioma have made some progress, the 5-year OS for glioma patients is only 10% (Buckner et al., 2017; Reifenberger et al., 2017). Thus, exploring novel and efficient diagnosis and therapeutic strategies is urgent. As a typical RNA modification, m1A has become a hot research issue. Recent studies have revealed that the alteration of m1A regulators is closely associated with multiple tumors including hepatocarcinoma, colon cancer, pancreatic cancer, cervical cancer, and glioma. Shi et al. (2020), Zheng et al. (2021) found that the expression of m1A regulators has great prognostic value in hepatocarcinoma and pancreatic cancer (Shi et al., 2020; Zheng et al., 2021). Gao et al. (2021) indicated that m1A regulator–mediated modification patterns play a crucial role in tumor microenvironment–infiltrating immune cells and the prognosis of colon cancer (Gao et al., 2021). Furthermore, one study demonstrated that the activation of TRM6/61 could promote tumor malignant progression via sustain tRNA in methylation status in glioma (Macari et al., 2016). However, the potential biological functions, and clinical and prognostic value of m1A regulators in glioma remain unclear.

Our study systematically analyzed the expression signatures, and clinical and prognostic values of m1A regulators in glioma for the first time, utilizing data extracted from the public databases. We found that ALKBH1, TRMT6, TRMT10C, YTHDF1, and YTHDF2 were significantly overexpressed in high-grade gliomas and the expression of TRMT6 and YTHDF2 was higher in IDH wild-type patients. Additionally, YTHDC1 was dramatically decreased in high-grade and IDH wild-type patients. Meanwhile ALKBH1, TRMT6, and YTHDF2 were significantly upregulated in 1p19q non-codel patients. Then we found that overexpression of TRMT61B, TRMT6, TRMT61A, YTHDFs, ALKBH1, and ALKBH3 was significantly associated with poor overall survival in glioma. Meanwhile, YTHDC1 was a potential protective factor in glioma. Consistently, YTHDF2 has been reported to act as an oncogene in numerous tumors, including glioma (Dixit et al., 2021; Jiang et al., 2021). The eraser ALKBH1 promotes cancer cell proliferation in glioma (Xie et al., 2018). And the overexpression of ALKBH3 could inhibit glioma cells death caused by D-2-HG. The reader YTHDC1 was reported as a suppressor gene in pancreatic cancer but an oncogene in acute myelocytic leukemia (Cheng et al., 2021; Hou et al., 2021). Macari et al. (2016) found that the activation of TRMT6/61 would promote malignant transformation and progression via sustain tRNA in methylation status in glioma (Macari et al., 2016). In summary, dysregulated m1A regulators may play a key role in the prognosis and progression of glioma.

Taking all the previous results together, we found that high expression of YTHDF2 and TRMT6 is closely correlated with advanced WHO stage, IDH mutation, 1p19q non-codel, and poor clinical outcome in glioma. Many studies have demonstrated that overexpression of YTHDF2 was significantly associated with higher malignant grades and a worse prognosis in glioma. Meanwhile, *in vivo* and *in vitro* experiments have confirmed that upregulated YTHDF2 facilitated the malignant progression of glioma (Chai et al., 2021). However, until now, there has been only one report addressing the function of TRMT6 in glioma. Macari et al. (2016) demonstrated that the activation of TRM6/61 promotes glioma progression (Macari et al., 2016). Moreover, we found that there were high correlations among m1A regulators, in particular between YTHDF2 and TRMT6. High expression of TRMT6 was closely associated with worse prognosis in different glioma clinical subtypes such as WHO-grade subtypes, IDH mutation status, and tumor recurrence status. Univariate and multivariate Cox regression analyses showed that TRMT6 is an independent prognostic risk factor in glioma. Collectively, TRMT6 may play a key role in the prognosis of glioma.

TRMT6 is a member of the tRNA methyltransferase family, which is involved in the posttranslational modification that produces the modified nucleoside m1A in tRNAs, thus functioning as a “writer” of m1A modification (Oerum et al., 2017). To explore the role of TRMT6, we performed functional experiments *in vitro*. Results showed that TRMT6 silencing could meaningfully inhibit cell proliferation, migration, and invasion in glioma. These results suggest that TRMT6 may act as an oncogene in glioma. Then, systematic bioinformatics analysis showed that cell cycle, PI3K-AKT, TGF-beta, MTORC1, NOTCH, and MYC pathways were significantly enriched in high–TRMT6 expression patients. Consistently, many studies have demonstrated that the activation of the cell cycle, PI3K-AKT, MTORC1, NOTCH, and MYC pathways enhances the progression of glioma (Parmigiani et al., 2020; Colardo and Segatto, 2021; Hajj et al., 2021). Notably, previously published literature has shown that TGF-beta ligands and its receptors exhibited low expression in low-grade gliomas (Yamada et al., 1995). Therefore, TGF-beta pathway is not activated and could not suppress the proliferation in low-grade glioma cells. However, the TGF-beta pathway is significantly activated in malignant gliomas, and the activation of TGF-beta pathway was closely associated with malignant progression and poor prognosis (Roy et al., 2015). The experimental study demonstrated that PCBP2 could activate TGF-beta pathway, thus promoting the development and progression of glioma (Mao et al., 2020). In summary, the aforementioned results showed that TRMT6 may be involved in glioma progression by regulating cell cycle, PI3K-AKT, TGF-beta, MTORC1, NOTCH, and MYC pathways. However, more detailed mechanisms need to be further explored.

## Conclusion

We proved the close relationship between the variation in m1A regulators and malignant tumor progression in glioma. We found that silencing TRMT6 suppressed the proliferation, migration, and invasion of glioma cells. These results present that m1A regulators, especially TRMT6, might play an essential role in the malignant progression of glioma.

## Data Availability

Publicly available datasets were analyzed in this study. These data can be found as follows: The RNA-seq transcriptome data and corresponding clinicopathologic information were downloaded from The Cancer Genome Atlas (TCGA, https://tcga-data.nci.nih.gov/tcga) and the Chinese Glioma Genome Atlas (CGGA, http://www.cgga.org.cn), and the immunohistochemistry of m1A regulators in normal and glioma tissues by Human Protein Atlas (HPA, http://www.proteinatlas.org).

## References

[B1] Brandsma D. Stalpers L. Taal W. Sminia P. van den Bent M. J. (2008). Clinical Features, Mechanisms, and Management of Pseudoprogression in Malignant Gliomas. Lancet Oncol. 9 (5), 453–461. 10.1016/s1470-2045(08)70125-6 18452856

[B2] Brown T. J. Brennan M. C. Li M. Church E. W. Brandmeir N. J. Rakszawski K. L. (2016). Association of the Extent of Resection with Survival in Glioblastoma. JAMA Oncol. 2 (11), 1460–1469. 10.1001/jamaoncol.2016.1373 27310651 PMC6438173

[B3] Buckner J. Giannini C. Eckel-Passow J. Lachance D. Parney I. Laack N. (2017). Management of Diffuse Low-Grade Gliomas in Adults - Use of Molecular Diagnostics. Nat. Rev. Neurol. 13 (6), 340–351. 10.1038/nrneurol.2017.54 28497806

[B4] Chai R.-C. Chang Y.-Z. Chang X. Pang B. An S. Y. Zhang K.-N. (2021). YTHDF2 Facilitates UBXN1 mRNA Decay by Recognizing METTL3-Mediated m6A Modification to Activate NF-Κb and Promote the Malignant Progression of Glioma. J. Hematol. Oncol. 14 (1), 109. 10.1186/s13045-021-01124-z 34246306 PMC8272379

[B5] Chen Z. Qi M. Shen B. Luo G. Wu Y. Li J. (2019). Transfer RNA Demethylase ALKBH3 Promotes Cancer Progression via Induction of tRNA-Derived Small RNAs. Nucleic Acids Res. 47 (5), 2533–2545. 10.1093/nar/gky1250 30541109 PMC6411830

[B6] Cheng Y. Xie W. Pickering B. F. Chu K. L. Savino A. M. Yang X. (2021). N6-Methyladenosine on mRNA Facilitates a Phase-Separated Nuclear Body that Suppresses Myeloid Leukemic Differentiation. Cancer Cell 39 (7), 958–972.e958. 10.1016/j.ccell.2021.04.017 34048709 PMC8282764

[B7] Chujo T. Suzuki T. (2012). Trmt61B Is a Methyltransferase Responsible for 1-methyladenosine at Position 58 of Human Mitochondrial tRNAs. Rna 18 (12), 2269–2276. 10.1261/rna.035600.112 23097428 PMC3504677

[B8] Colardo M. Segatto M. Di Bartolomeo S. (2021). Targeting RTK-Pi3k-mTOR Axis in Gliomas: An Update. Ijms 22 (9), 4899. 10.3390/ijms22094899 34063168 PMC8124221

[B9] Dai X. Wang T. Gonzalez G. Wang Y. (2018). Identification of YTH Domain-Containing Proteins as the Readers for N1-Methyladenosine in RNA. Anal. Chem. 90 (11), 6380–6384. 10.1021/acs.analchem.8b01703 29791134 PMC6157021

[B10] Dawson M. A. Kouzarides T. (2012). Cancer Epigenetics: from Mechanism to Therapy. Cell 150 (1), 12–27. 10.1016/j.cell.2012.06.013 22770212

[B11] Dixit D. Prager B. C. Gimple R. C. Poh H. X. Wang Y. Wu Q. (2021). The RNA m6A Reader YTHDF2 Maintains Oncogene Expression and Is a Targetable Dependency in Glioblastoma Stem Cells. Cancer Discov. 11 (2), 480–499. 10.1158/2159-8290.cd-20-0331 33023892 PMC8110214

[B12] Engel M. Chen A. (2018). The Emerging Role of mRNA Methylation in normal and Pathological Behavior. Genes, Brain Behav. 17 (3), e12428. 10.1111/gbb.12428 29027751

[B13] Gao Y. Wang H. Li H. Ye X. Xia Y. Yuan S. (2021). Integrated Analyses of m1A Regulator-Mediated Modification Patterns in Tumor Microenvironment-Infiltrating Immune Cells in colon Cancer. OncoImmunology 10 (1), 1936758. 10.1080/2162402x.2021.1936758 34221700 PMC8224220

[B14] Gilbert W. V. Bell T. A. Schaening C. (2016). Messenger RNA Modifications: Form, Distribution, and Function. Science 352 (6292), 1408–1412. 10.1126/science.aad8711 27313037 PMC5094196

[B15] Gusyatiner O. Hegi M. E. (2018). Glioma Epigenetics: From Subclassification to Novel Treatment Options. Semin. Cancer Biol. 51, 50–58. 10.1016/j.semcancer.2017.11.010 29170066

[B16] Hajj G. N. M. Nunes P. B. C. Roffe M. (2021). Genome-wide Translation Patterns in Gliomas: An Integrative View. Cell Signal. 79, 109883. 10.1016/j.cellsig.2020.109883 33321181

[B17] Hänzelmann S. Castelo R. Guinney J. (2013). GSVA: Gene Set Variation Analysis for Microarray and RNA-Seq Data. BMC Bioinformatics 14, 7. 10.1186/1471-2105-14-7 23323831 PMC3618321

[B18] Hou Y. Zhang Q. Pang W. Hou L. Liang Y. Han X. (2021). YTHDC1-mediated Augmentation of miR-30d in Repressing Pancreatic Tumorigenesis via Attenuation of RUNX1-Induced Transcriptional Activation of Warburg Effect. Cell Death Differ. 10.1038/s41418-021-00804-0 PMC8563797 34021267

[B19] Huang H. Weng H. Chen J. (2020). m6A Modification in Coding and Non-coding RNAs: Roles and Therapeutic Implications in Cancer. Cancer Cell 37 (3), 270–288. 10.1016/j.ccell.2020.02.004 32183948 PMC7141420

[B20] Jiang X. Liu B. Nie Z. Duan L. Xiong Q. Jin Z. (2021). The Role of m6A Modification in the Biological Functions and Diseases. Sig Transduct Target. Ther. 6 (1), 74. 10.1038/s41392-020-00450-x PMC7897327 33611339

[B21] Komal S. Zhang L.-R. Han S.-N. (2021). Potential Regulatory Role of Epigenetic RNA Methylation in Cardiovascular Diseases. Biomed. Pharmacother. 137, 111376. 10.1016/j.biopha.2021.111376 33588266

[B22] Lapointe S. Perry A. Butowski N. A. (2018). Primary Brain Tumours in Adults. The Lancet 392 (10145), 432–446. 10.1016/s0140-6736(18)30990-5 30060998

[B23] Macari F. El-Houfi Y. Boldina G. Xu H. Khoury-Hanna S. Ollier J. (2016). TRM6/61 Connects PKCα with Translational Control through tRNAiMet Stabilization: Impact on Tumorigenesis. Oncogene 35 (14), 1785–1796. 10.1038/onc.2015.244 26234676

[B24] Mao J. Sun Z. Cui Y. Du N. Guo H. Wei J. (2020). PCBP2 Promotes the Development of Glioma by Regulating FHL3/TGF‐β/Smad Signaling Pathway. J. Cel Physiol 235 (4), 3280–3291. 10.1002/jcp.29104 PMC7166520 31693182

[B25] Oerum S. Dégut C. Barraud P. Tisné C. (2017). m1A Post‐Transcriptional Modification in tRNAs. Biomolecules 7 (1), 20. 10.3390/biom7010020 PMC5372732 28230814

[B26] Parmigiani E. Taylor V. Giachino C. (2020). Oncogenic and Tumor-Suppressive Functions of NOTCH Signaling in Glioma. Cells 9 (10), 2304. 10.3390/cells9102304 PMC7602630 33076453

[B27] Reifenberger G. Wirsching H.-G. Knobbe-Thomsen C. B. Weller M. (2017). Advances in the Molecular Genetics of Gliomas - Implications for Classification and Therapy. Nat. Rev. Clin. Oncol. 14 (7), 434–452. 10.1038/nrclinonc.2016.204 28031556

[B28] Roy L.-O. Poirier M.-B. Fortin D. (2015). Transforming Growth Factor-Beta and its Implication in the Malignancy of Gliomas. Targ Oncol. 10 (1), 1–14. 10.1007/s11523-014-0308-y 24590691

[B29] Safra M. Sas-Chen A. Nir R. Winkler R. Nachshon A. Bar-Yaacov D. (2017). The m1A Landscape on Cytosolic and Mitochondrial mRNA at Single-Base Resolution. Nature 551 (7679), 251–255. 10.1038/nature24456 29072297

[B30] Shi H. Chai P. Jia R. Fan X. (2020). Novel Insight into the Regulatory Roles of Diverse RNA Modifications: Re-defining the Bridge between Transcription and Translation. Mol. Cancer 19 (1), 78. 10.1186/s12943-020-01194-6 32303268 PMC7164178

[B31] Subramanian A. Tamayo P. Mootha V. K. Mukherjee S. Ebert B. L. Gillette M. A. (2005). Gene Set Enrichment Analysis: a Knowledge-Based Approach for Interpreting Genome-wide Expression Profiles. Proc. Natl. Acad. Sci. 102 (43), 15545–15550. 10.1073/pnas.0506580102 16199517 PMC1239896

[B32] Swietlik E. M. Ghataorhe P. Zalewska K. I. Wharton J. Howard L. S. Taboada D. (2021). Plasma Metabolomics Exhibit Response to Therapy in Chronic Thromboembolic Pulmonary Hypertension. Eur. Respir. J. 57 (4), 2003201. 10.1183/13993003.03201-2020 33060150 PMC8012591

[B33] Tasaki M. Shimada K. Kimura H. Tsujikawa K. Konishi N. (2011). ALKBH3, a Human AlkB Homologue, Contributes to Cell Survival in Human Non-small-cell Lung Cancer. Br. J. Cancer 104 (4), 700–706. 10.1038/sj.bjc.6606012 21285982 PMC3049579

[B34] Vissers C. Sinha A. Ming G.-l. Song H. (2020). The Epitranscriptome in Stem Cell Biology and Neural Development. Neurobiol. Dis. 146, 105139. 10.1016/j.nbd.2020.105139 33065280 PMC7686257

[B35] Wang Y. Huang Q. Deng T. Li B.-H. Ren X.-Q. (2019). Clinical Significance of TRMT6 in Hepatocellular Carcinoma: A Bioinformatics-Based Study. Med. Sci. Monit. 25, 3894–3901. 10.12659/msm.913556 31128068 PMC6556066

[B36] Xie Q. Wu T. P. Gimple R. C. Li Z. Prager B. C. Wu Q. (2018). N-methyladenine DNA Modification in Glioblastoma. Cell 175 (5), 1228–1243.e1220. 10.1016/j.cell.2018.10.006 30392959 PMC6433469

[B37] Xiong X. Li X. Yi C. (2018). N1-methyladenosine Methylome in Messenger RNA and Non-coding RNA. Curr. Opin. Chem. Biol. 45, 179–186. 10.1016/j.cbpa.2018.06.017 30007213

[B38] Yamada N. Kato M. Yamashita H. Nistér M. Miyazono K. Heldin C.-H. (1995). Enhanced Expression of Transforming Growth Factor-β and its Type-I and Type-II Receptors in Human Glioblastoma. Int. J. Cancer 62 (4), 386–392. 10.1002/ijc.2910620405 7635563

[B39] Zhang C. Jia G. (2018). Reversible RNA Modification N 1 -methyladenosine (M 1 A) in mRNA and tRNA. Genomics, Proteomics & Bioinformatics 16 (3), 155–161. 10.1016/j.gpb.2018.03.003 PMC6076376 29908293

[B40] Zhao B. S. Roundtree I. A. He C. (2017). Post-transcriptional Gene Regulation by mRNA Modifications. Nat. Rev. Mol. Cel Biol 18 (1), 31–42. 10.1038/nrm.2016.132 PMC5167638 27808276

[B41] Zhao Y. Zhao Q. Kaboli P. J. Shen J. Li M. Wu X. (2019). m1A Regulated Genes Modulate PI3K/AKT/mTOR and ErbB Pathways in Gastrointestinal Cancer. Translational Oncol. 12 (10), 1323–1333. 10.1016/j.tranon.2019.06.007 PMC6661385 31352195

[B42] Zheng Q. Yu X. Zhang Q. He Y. Guo W. (2021). Genetic Characteristics and Prognostic Implications of m1A Regulators in Pancreatic Cancer. Biosci. Rep. 41 (4). 10.1042/bsr20210337 PMC8035622 33779693

