# Supplementary material for: RNA m1A Methyltransferase TRMT6 Predicts Poorer Prognosis and Promotes Malignant Behavior in Glioma
Source: Front Mol Biosci. 2021 Sep 22;8:692130. doi: 10.3389/fmolb.2021.692130 (PMC8493077; doi:10.3389/fmolb.2021.692130)
Supplement: Supplementary file 1 [file DataSheet1.PDF]

Supplementary materials

Table S1. Information of datasets used in this study.

Datasets	Platforms	Non-tumor samples	Tumor samples	Year	Country
CGGA	Illumina	0	693	2015	China
TCGA	Illumina	5	668	2017	USA
**Total**		**5**	**1361**		

Table S2. Complete list of primers and siRNA sequence used in this work.

Name	sequence
TRMT6	Forward: 5’TTCAAGGCCGTTTGTGGTCT3’<br>Reverse: 5’CCACCTCCACTCATCAGCAG3’
TRMT6 siRNA1	SS Sequence GGGUGUUGUUAGAAGGCAAGG<br>AS Sequence UAGUGUAUCGUAUCUCAUGUG
TRMT6 siRNA2	SS Sequence AGAUCGAAGUCAUCCUAAACU<br>AS Sequence UUUAGGAUGACUUCGAUCUGG
TRMT6 siRNA3	SS Sequence CGCAGAUGGUUUAAUUGUAGC<br>AS Sequence UACAAUUAAACCAUCUGCGUU

20 × 20

FigureS1: The significant differential expression m1A regulators stratified by 1p19q status in gliomas based on TCGA and CGGA datasets.

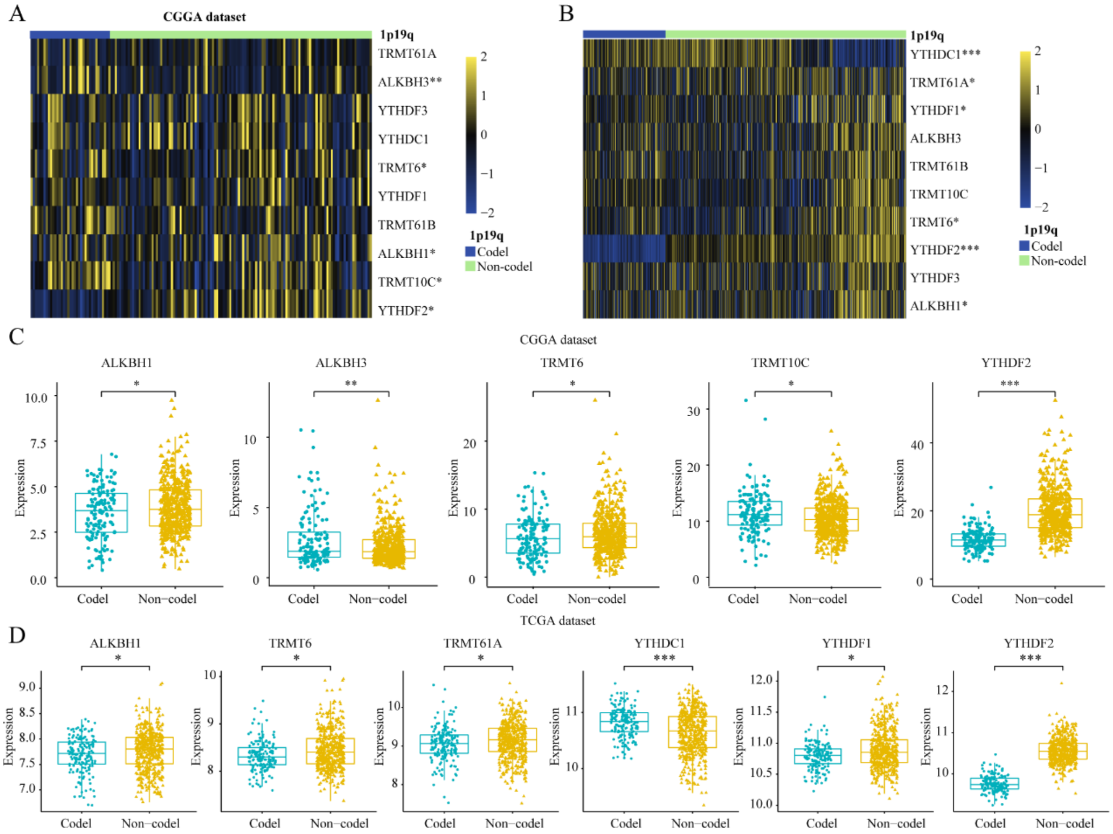

(A-B) The differential expression of m1A regulators stratified by 1p19q codeletion statuses in glioma according to TCGA and CGGA databases. *P < 0.05, **P < 0.01, ***P < 0.001.

20 × 20

FigureS2: m1A regulators protein expression level in non-tumor tissues and glioma tissues.

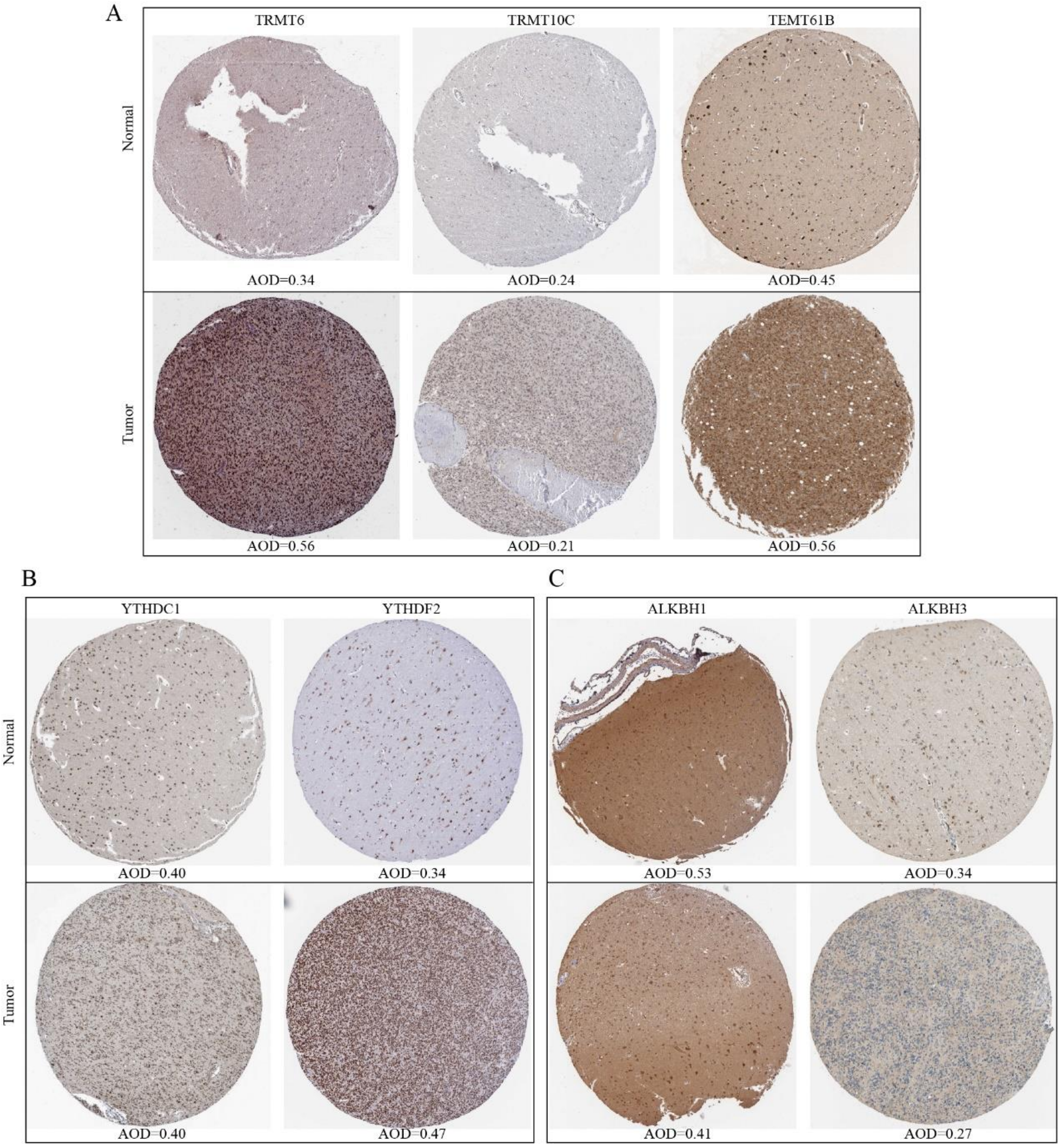

20 × 20

FigureS3: Correlation analysis of YTHDF2 and TRMT6 and their prognostic value in glioma based on TCGA and CGGA datasets.

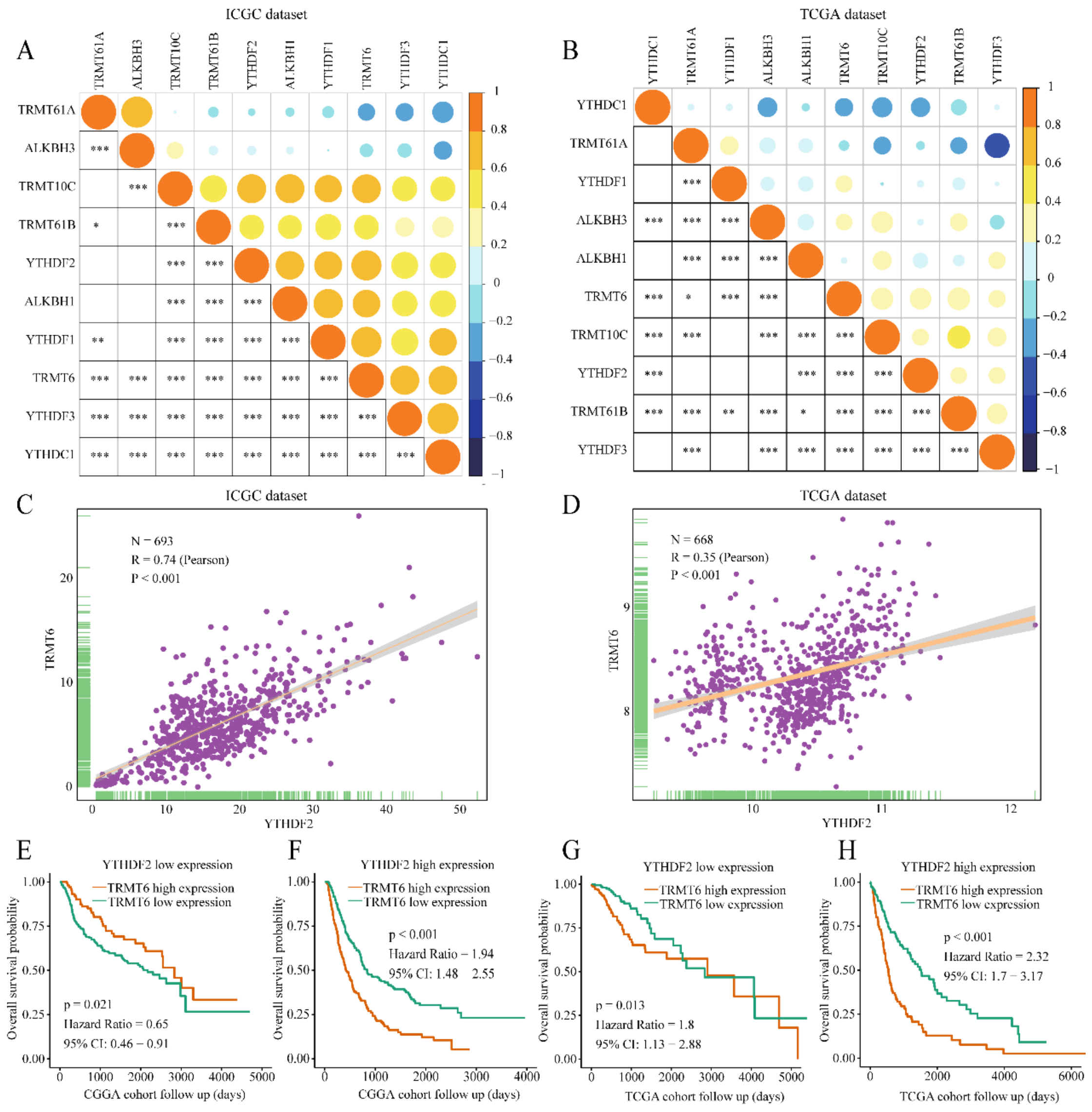

(A-B) Pearson correlation analysis of the 10 m1A regulators. **(C-D)** Correlation analysis of YTHDF2 and TRMT6. **(E-F)** Kaplan-Meier overall survival curves of TRMT6 for patients stratified by YTHDF2 expression level.

20 × 20

FigureS4: Original microscopic pictures of Clone formation and transwell experiments.

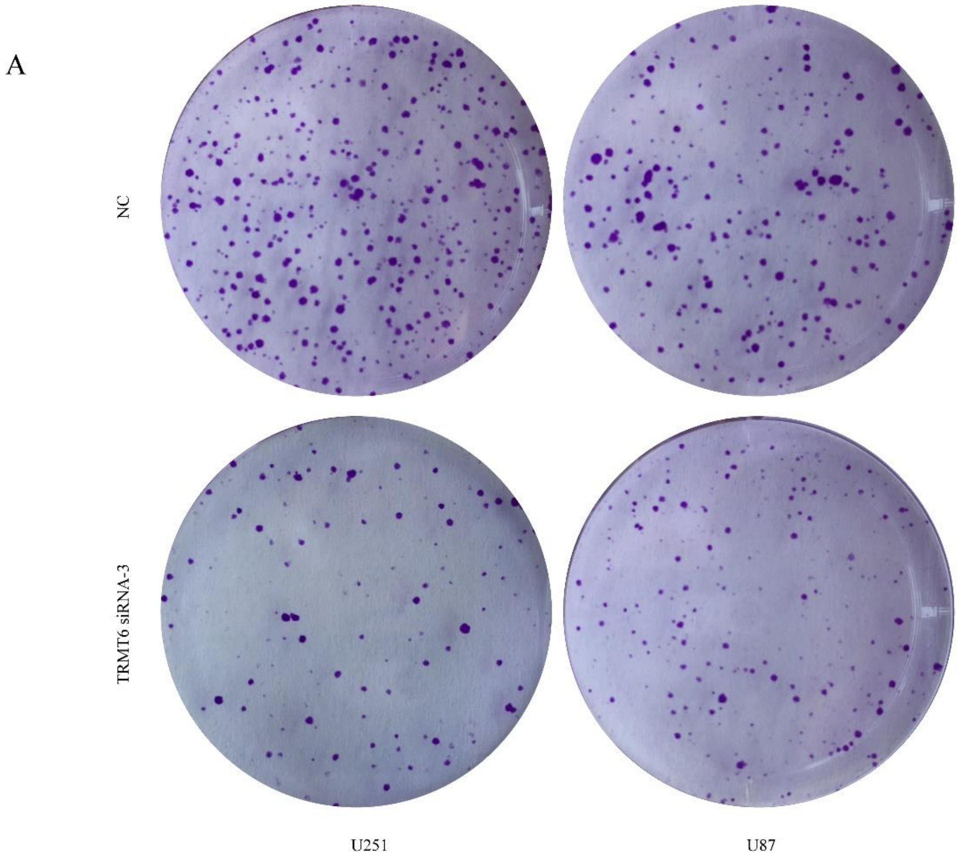

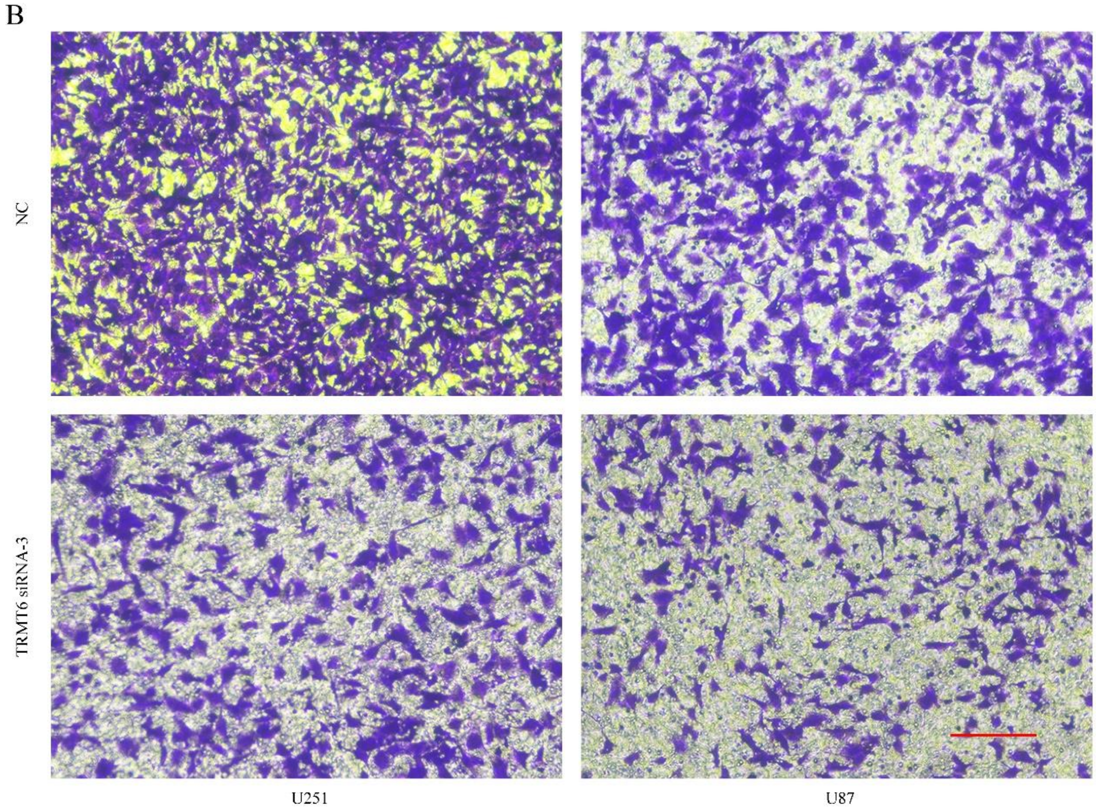

20 × 20

FigureS4: Original microscopic pictures of wound scratch and EdU assays.

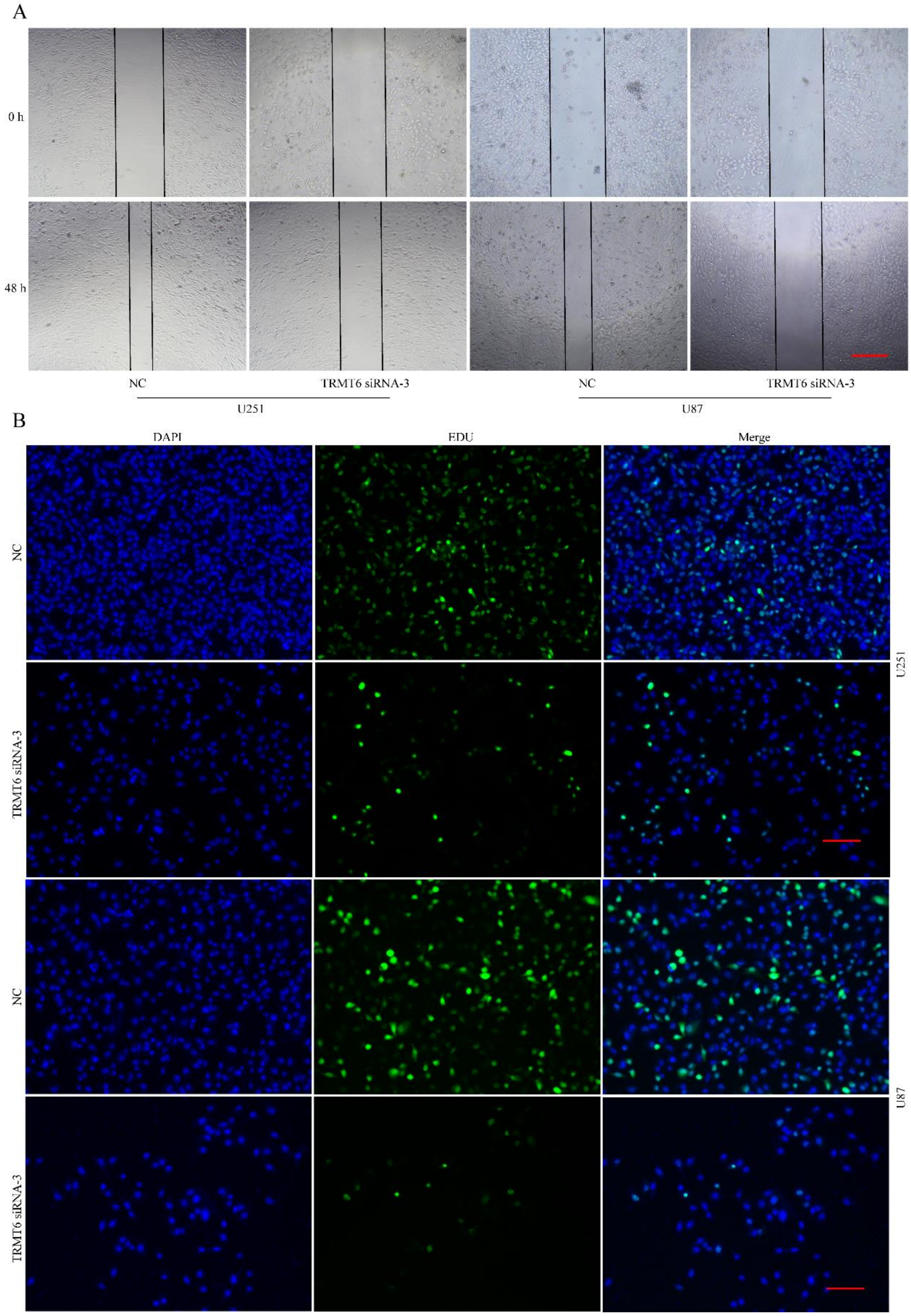

20 × 20
